# Designing molecular diagnostics for current tuberculosis drug regimens

**DOI:** 10.1080/22221751.2023.2178243

**Published:** 2023-02-27

**Authors:** Sophia B. Georghiou, Margaretha de Vos, Kavindhran Velen, Paolo Miotto, Rebecca E. Colman, Daniela Maria Cirillo, Nazir Ismail, Timothy C. Rodwell, Anita Suresh, Morten Ruhwald

**Affiliations:** a FIND, the Global Alliance for Diagnostics, Geneva, Switzerland; b IRCCS San Raffaele Scientific Institute, Milan, Italy; c Department of Medicine, University of California, San Diego, La Jolla, CA, USA; d World Health Organization, Geneva, Switzerland

**Keywords:** Bedaquiline, nitroimidazoles, linezolid, pyrazinamide, molecular diagnostics

## Abstract

Diagnostic development must occur in parallel with drug development to ensure the longevity of new treatment compounds. Despite an increasing number of novel and repurposed anti-tuberculosis compounds and regimens, there remains a large number of drugs for which no rapid and accurate molecular diagnostic option exists. The lack of rapid drug susceptibility testing for linezolid, bedaquiline, clofazimine, the nitroimidazoles (i.e pretomanid and delamanid) and pyrazinamide at any level of the healthcare system compromises the effectiveness of current tuberculosis and drug-resistant tuberculosis treatment regimens. In the context of current WHO tuberculosis treatment guidelines as well as promising new regimens, we identify the key diagnostic gaps for initial and follow-on tests to diagnose emerging drug resistance and aid in regimen selection. Additionally, we comment on potential gene targets for inclusion in rapid molecular drug susceptibility assays and sequencing assays for novel and repurposed drug compounds currently prioritized in current regimens, and evaluate the feasibility of mutation detection given the design of existing technologies. Based on current knowledge, we also propose design priorities for next generation molecular assays to support triage of tuberculosis patients to appropriate and effective treatment regimens. We encourage assay developers to prioritize development of these key molecular assays and support the continued evolution, uptake, and utility of sequencing to build knowledge of tuberculosis resistance mechanisms and further inform rapid treatment decisions in order to curb resistance to critical drugs in current regimens and achieve End TB targets.

**Trial registration:** ClinicalTrials.gov identifier: NCT05117788..

## Background

Novel tuberculosis (TB) treatment regimens have the potential to accelerate the World Health Organization (WHO) End TB efforts, with an increasing number of recommended new and repurposed compounds [1, 2]. These new regimens are generally safer, shorter, and better tolerated by patients, marking a landmark for TB patient care. However, the efficacy of these regimens remains dependent on evidence-based clinical decision-making, specifically, detection and/or rule-out of resistance to key drug compounds prior to initiating treatment.

**Figure 1.**
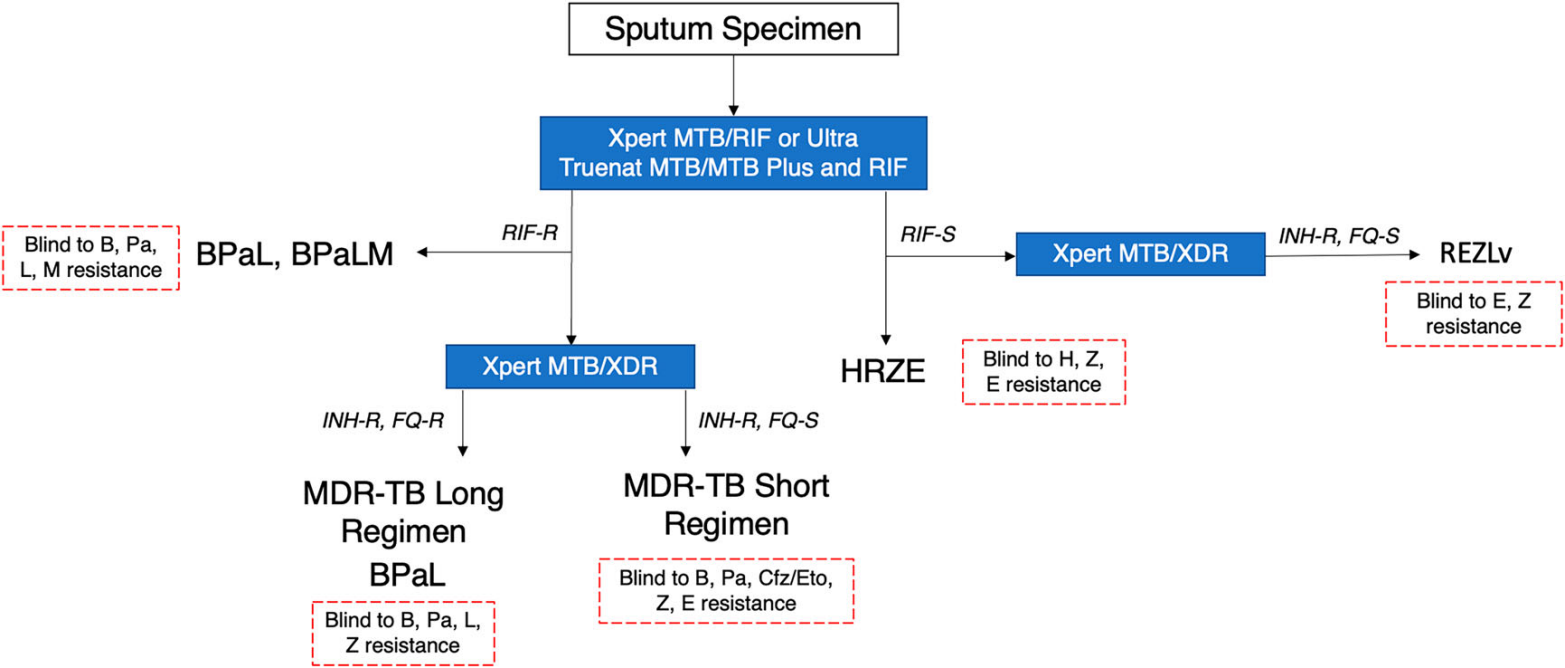
Example of a molecular diagnostic cascade for programmatic management of drug-resistant tuberculosis at lower-level healthcare centres. Note: Lower-level health centres refer to intermediate and peripheral laboratories with limited or minimal infrastructure where technicians with adequate training can perform routine diagnostic testing with molecular tests in 1-2 h [64]. Samples may also be referred to centralized laboratories for additional testing (e.g. additional molecular DST of rifampicin-resistant patients) given rapid and safe transport of specimens from health facilities or lower-level laboratories to the higher-level laboratory, as well as expedient reporting of results back to clinicians. Rapid molecular diagnostic testing of treatment non-responders must also be considered following regimen selection and treatment initiation (e.g. month 2 following treatment initiation) to determine acquired resistance. It should be noted that intermediate centres may also have access to certain instruments such as BD, Abbott or Roche systems, as in Figure 2. B, bedaquiline; Cfz, clofazimine; E, ethambutol; Eto, ethionamide; H, isoniazid; L, linezolid; Lv, levofloxacin; Pa, pretomanid; R, rifampicin; Z, pyrazinamide.

**Figure 2.**
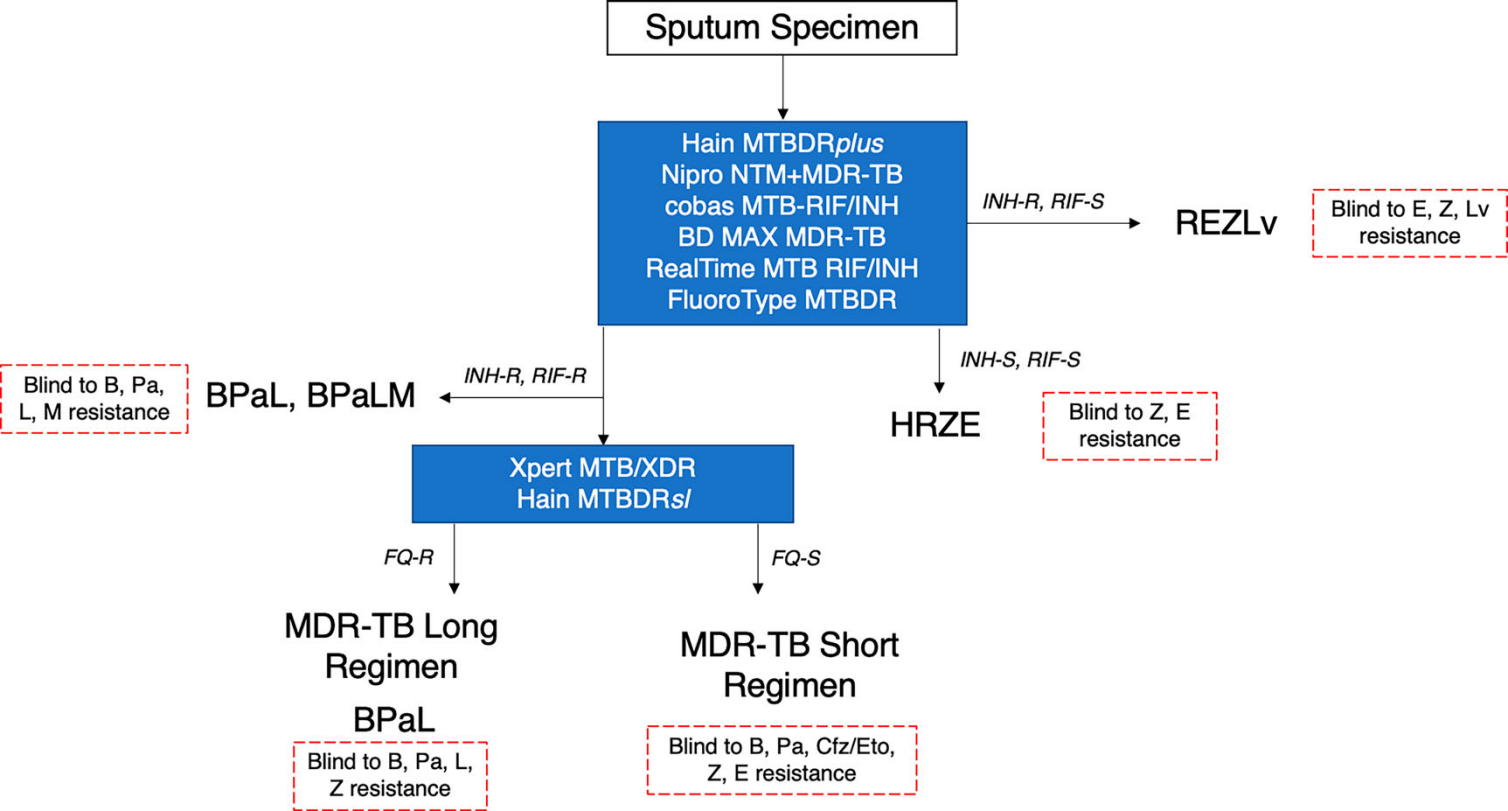
Example of a molecular diagnostic cascade for higher-level health centres. Note: Higher-level health centres refer to reference laboratories with sufficient infrastructure as well as well-established laboratory networks and trained personnel to run higher-throughput and complex molecular tests [64]. Rapid molecular diagnostic testing of treatment non-responders must also be considered following regimen selection and treatment initiation (e.g. month 2 following treatment initiation) to determine acquired resistance. The Nipro PZA LPA might also be used at any point along the cascade to evaluate pyrazinamide resistance. B, bedaquiline; Cfz, clofazimine; E, ethambutol; Eto, ethionamide; H, isoniazid; L, linezolid; Lv, levofloxacin; Pa, pretomanid; R, rifampicin; Z, pyrazinamide

In order to preserve new drugs as TB treatment options, there is a critical need to rapidly identify resistance to these compounds. However, TB drug and diagnostic development pathways are not aligned, and there is often a significant lag time between which regimens are approved for use and clinical resistance is detected, with production of novel diagnostics only triggered following detection of resistance to regimens in clinical use. Currently, no WHO-endorsed molecular diagnostics currently exist to rapidly detect resistance to many new anti-TB compounds (Table 1). In lieu of rapid solutions for expanded resistance detection, clinicians must rely on phenotypic drug susceptibility testing (pDST) for new and repurposed drugs. When available, pDST often reveals important information too late, leaving most patients to be treated empirically with compounds to which they might already be resistant, with resistance already noted to some of the most important novel compounds in our TB drug arsenal [3-8]. Ultimately, for effective patient management and reductions in TB incidence, new drug compounds must be used in parallel with appropriate, rapid and accurate diagnostics that inform treatment in both lower- and higher-level healthcare centres (Figures 1 and 2).

**Table 1.** Current WHO-endorsed diagnostic options and technologies under development or evaluation for tuberculosis drug resistance detection stratified by drug class.

Treatment	Drug	Low complexity automated NAATs	Moderate complexity automated NAATs	High complexity reverse hybridization-based NAATs	Targeted NGS	MICs	pDST
First-line	R	TruenatRIF; Xpert MTB/RIF; Xpert MTB/RIF Ultra; Bioneer IRON-qPCR RFIA Kit*; SD Biosensor STANDARD M MDR-TB*	Abbott RealTi*m*e MTB RIF/INH; FluoroType MTBDR; BD MAX MDR-TB; cobas MTB-RIF/INH; Zeesan MeltPro MTB/RIF*; Seegene Anyplex II MTB/MDR/XDR Detection assay*	GenoType MTBDR*plus;* Nipro Genoscholar NTM + MDRTB Detection Kit	Genoscreen Deeplex*	Sensititre MYCOTB*; CRyPTIC UKMYC5*	LJ; 7H10; 7H11; MGIT
	H**	Xpert MTB/XDR; Bioneer IRON-qPCR RFIA Kit*; SD Biosensor STANDARD M MDR-TB*	Abbott RealTi*m*e MTB RIF/INH; FluoroType MTBDR; BD MAX MDR-TB; cobas MTB-RIF/INH; Zeesan MeltPro MTB/INH*; Seegene Anyplex II MTB/MDR/XDR Detection assay*	GenoType MTBDR*plus;* Nipro Genoscholar NTM + MDRTB Detection Kit	Genoscreen Deeplex*	Sensititre MYCOTB*; CRyPTIC UKMYC5*	LJ; 7H10; 7H11; MGIT
	E		FluoroType XDR*; Zeesan MeltPro MTB/EMB*		Genoscreen Deeplex*	CRyPTIC	LJ; 7H10; 7H11; MGIT
						UKMYC5*	
	Z			Nipro Genoscholar PZA-TB II	Genoscreen Deeplex*		MGIT
Group A	FQ	Xpert MTB/XDR; Bioneer IRON-qPCR RFIA Kit*	FluoroType XDR*; Zeesan MeltPro MTB/FQ*; Seegene Anyplex II MTB/MDR/XDR Detection assay*	GenoType MTBDR*sl*	Genoscreen Deeplex*	Sensititre MYCOTB*; CRyPTIC UKMYC5*	LJ; 7H10; 7H11; MGIT
	B				Genoscreen Deeplex*		7H11; MGIT
	L		FluoroType XDR*		Genoscreen Deeplex*	CRyPTIC UKMYC5*	7H10; 7H11; MGIT
Group B	Cfz				Genoscreen Deeplex*	CRyPTIC UKMYC5*	MGIT
	DCS					Sensititre MYCOTB*	
Group C	DLM					CRyPTIC UKMYC5*	7H11; MGIT
	IMP-CLN/MPM						
	AMK	Xpert MTB/XDR; Bioneer IRON-qPCR RFIA Kit*	FluoroType XDR*; Zeesan MeltPro MTB/SL*; Seegene Anyplex II MTB/MDR/XDR Detection assay*	GenoType MTBDR*sl*	Genoscreen Deeplex*	Sensititre MYCOTB*; CRyPTIC UKMYC5*	LJ; 7H10; MGIT
	STR		Zeesan MeltPro MTB/STR*		Genoscreen Deeplex*	Sensititre MYCOTB*	LJ; 7H10; 7H11; MGIT
	ETO/Pa	Xpert MTB/XDR			Genoscreen Deeplex*	Sensititre MYCOTB*; CRyPTIC UKMYC5*	LJ; 7H10; 7H11; MGIT
	PAS					Sensititre MYCOTB*; CRyPTIC UKMYC5*	

AMK, amikacin; B, bedaquiline; Cfz, clofazimine; DCS, D-cycloserine; DLM, delamanid; E, ethambutol; ETO, ethionamide; FQ. fluoroquinolones; H, isoniazid; IMP-CLN, imipenem-cilastatin; L, linezolid; MPM, meropenem; NAAT, nucleic acid amplification test; Pa, pretomanid; PAS, para-aminosalicyclic acid; pDST, phenotypic drug susceptibility testing; R, rifampicin; STR, streptomycin; Z, pyrazinamide.

*Additional technologies without WHO recommendation or review to date [64], but known to be under development or evaluation for TB drug resistance detection [37-84], are also included in the table. SD Biosensor is additionally developing an assay for detection of mutations associated with resistance to pre-XDR and/or XDR drugs, though the specific drugs to be included in the assay were not confirmed.

**Most molecular assays for H resistance detection cover at least one gene target (i.e. *inhA* promoter) also associated with ETO resistance, and so resistance to this drug might be inferred.

In this regard, the release of the 2021 WHO TB catalogue of mutations associated with drug resistance was a landmark for the field, guiding diagnostic developers on priority targets and interpretation of mutations associated with phenotypic resistance [9]. In parallel, minimum inhibitory concentration (MIC)-based DST through microtiter plates continues to add valuable knowledge to the field [10, 11]. However, the association between genetic targets and the expression of clinically relevant phenotypic resistance remains less well understood for the newer drugs, and studies to date have generally revealed a complicated genetic landscape of resistance, highlighting the need for new approaches to resistance diagnosis. Although Next Generation Sequencing (NGS) approaches cover a diverse array of complex genetic targets to “rule-in” and/or “rule-out” resistance, workflows are still complex and only suitable for centralized testing. While culture-free, targeted NGS (tNGS) solutions eliminate the need for the biosafety level 3 facilities required for pDST, the multi-step workflows and high level of training required for implementation limits their broader use outside of the laboratory, leaving important gaps at point-of-care for diagnosis of drug-resistant (DR-)TB patients.

Based upon the expanding knowledge base of molecular mechanisms of important drug resistance, and in the context of current TB treatment regimens, we identify key gaps in existing tests to diagnose clinically relevant resistance at all levels of the healthcare system, while considering the potential for resistance development and the risk of treatment failure if resistance is not diagnosed. Additionally, we suggest appropriate designs for rapid molecular drug susceptibility assays for novel drug compounds, focusing on linezolid, bedaquiline, the nitroimidazoles (i.e. pretomanid and delamanid) and pyrazinamide, considering both “rule-in” and “rule-out” resistance assays with the potential to fill these gaps and improve TB patient diagnosis and treatment worldwide.

## Bedaquiline

Bedaquiline has been shown to greatly improve DR-TB patient outcomes in both clinical trials and TB programmes [12, 13]. In 2019, WHO conditionally recommended bedaquiline in both shorter and longer rifampicin-resistant/multidrug-resistant (MDR)-TB regimens (Figures 1 and 2) [1], and more recently recommended the 6-month BPaLM regimen for treatment of patients with MDR-TB, and BPaL for MDR-TB patients with documented fluoroquinolone resistance [2]. Notably, bedaquiline DST is also a minimal requirement for next-generation molecular DST at peripheral centres [14]. Additional, ongoing evaluations of bedaquiline-containing regimens, including BPaMZ, are likely to add to the current evidence base and recommendations to improve TB patient treatment.

### Drug resistance

Despite the importance of bedaquiline in various regimens, programmatic implementation efforts have been challenged by the length and safety of these regimens, which are variables that contribute to resistance development [15]. Bedaquiline notably has a long half-life, posing a risk for resistance acquisition after therapy discontinuation or if used with drugs with mismatched half-lives [16]. Although overall rates are low [17], elevated bedaquiline MICs have also been documented in isolates from patients without prior bedaquiline exposure, suggesting potential cross-resistance mechanisms and/or that natural resistance might already exist in certain regions, especially for specific *M. tuberculosis* lineages [18, 19]. The risk of treatment failure is likely high for patients in which resistance is not diagnosed prior to bedaquiline treatment [20, 21], as seen in the ZeNix BPaL trial, with 3/9 (33%) participants with baseline phenotypic bedaquiline resistance having unfavourable outcomes [22]. To date, however, only pDST methods exist to determine resistance [23].

The mechanisms of bedaquiline resistance are complex, involving long stretches of several genes that harbour a range of mutations including neutral polymorphisms, making a targeted nucleic acid amplification test (NAAT) approach difficult. Bedaquiline resistance has been associated with mutations in *atpE*, *pepQ*, *mmpR*, and possibly *Rv1979* [19], with *pepQ*, *mmpR* and *Rv1979* mutations also implicated in cross-resistance to clofazimine [24–29]. *pepQ* and *mmpR* would be particularly challenging genes for inclusion in NAATs as these genes are non-essential, so there is a spectrum of mutations including neutral polymorphisms which must be excluded or ignored bioinformatically.

In the 2021 WHO mutations catalog, no mutations met the established confidence-grading criteria for association with bedaquiline – (or clofazimine-) resistant phenotypes, mostly due to the very small number of bedaquiline-resistant organisms in the collection [9]. The next version of the catalog will include additional data from additional bedaquiline-resistant organisms and establish select variants as confidence-graded markers of phenotypic bedaquiline resistance [30]. The most reported resistance mutations from the literature are in genes *atpE* and *mmpR*, making them priority targets for NAATs. Of the *atpE* mutations, only A63P appears to be a resistance marker with potential clinical relevance [31, 32]. Collectively, *atpE* mutations only appear to be responsible for a small portion of phenotypic bedaquiline resistance documented globally [33]. One explanation is that *atpE* mutants may be associated with lower fitness and are thus outcompeted by other mutants during treatment [4, 33]. The *mmpR* gene, in contrast, appears to be a more globally and clinically relevant genetic target, with a large number of *mmpR* mutations associated with both phenotypic bedaquiline resistance and unfavourable clinical outcomes [34, 35]. Based upon recent reviews including evidence from clinical mutants, *mmpR* frameshifts at nucleotides 192–199 and *atpE* A63P/V appear to be the most frequent markers associated with phenotypic bedaquiline resistance across multiple studies [31, 32]. These markers may be considered initial targets for assay inclusion (Supplement), though overall sample sizes are low, and the strength of marker association with phenotypic resistance can be variable due to the dynamics (i.e. exposure/activation) of the associated efflux pumps [5].

### Diagnostic assay design considerations

Apart from NGS approaches, which are currently limited to central laboratories, most molecular assays with field-deployable capabilities have a limited capacity for mutation detection, relying upon the detection of relatively short mutation “hotspots” for resistance determination. Bedaquiline is thus one of the most difficult drug targets to include in NAATs given the length of the associated resistance genes, the number of polymorphisms to be detected, and the presence of non-resistance conferring mutations that need to be disregarded. Nonetheless, bedaquiline represents one of the most important new drugs given its documented efficacy in all-oral DR-TB regimens [2, 12, 13], and this drug is likely to only grow in importance and utility given its important role in regimens currently undergoing trials. For this reason, an assay to detect bedaquiline resistance would have great value to multiple regimens.

Given the molecular complexities of predicting phenotypic bedaquiline resistance, it would benefit manufacturers to prioritize assay designs that cover large swaths of multiple genes, such as amplifying whole genes as appropriate for certain line probe assays and real-time PCR assays and adding new mutation detection probes as confidence grows in specific resistance markers. NGS solutions are particularly well-suited to such a broad and inclusive approach, with the added ability to bioinformatically display or conceal specific resistance mutations as evidence of their clinical relevance builds. Although any assay revision would ultimately require additional data generation to support performance review and regulatory approval, the ability to quickly revise open-format assays would at least preclude the need for expensive and time-consuming redesign and ultimately lower the time to generation of performance evidence for the updated assays. Furthermore, if the assay already covers specific variants and only the resistance calling algorithms are changed or re-trained, it may be possible to use existing databases to generate performance evidence. In regards to specific targets, the inclusion of *mmpR* mutations in at least the 192–199 gene region in addition to *atpE* A63P/V in a more targeted NAAT could already serve to “rule in” resistance in this regard, similar to how ethionamide resistance detection is currently handled by Xpert MTB/XDR [36]. Such an assay would be expected to have an impact on clinical decision making, despite expected performance limitations from an overall sensitivity perspective.

With respect to these limitations, another suitable approach for a bedaquiline resistance assay would be to “rule out” resistance by screening for the absence of relevant mutations, deletions or frameshifts. The absence of any genetic variant in *atpE* and *mmpR*, in the context of the current low prevalence of these mutations in clinical settings, would likely yield substantial negative predictive values and have important clinical implications for treatment. Some sequencing assays are already designed to serve this function and effectively “rule out” resistance [37], and might be scaled up for this purpose in lieu of the availability of field-deployable NAATs. Ultimately, additional studies correlating mutation and pDST data for a larger selection of isolates, as underway through WHO and elsewhere [38], are critically needed to further inform design and interpretation of molecular diagnostics for bedaquiline resistance detection.

## Linezolid

Linezolid is an oxazolidinone antibiotic that exhibits bacteriostatic activity against *M. tuberculosis* complex by disrupting protein synthesis [39]. The fact that linezolid can be taken orally is a distinct advantage for TB treatment, providing a valuable alternative to long-term daily injections, with associated improvements in patient treatment outcomes [13]. WHO has strongly recommended linezolid as a "Group A” medicine for the longer rifampicin-resistant/MDR-TB regimen, and recently recommended the 6-month BPaL and BPaLM regimens for treatment of MDR-TB patients (Figures 1 and 2) [1, 2].

### Drug resistance

The threat of clinically relevant bacterial resistance to linezolid is a concern given that optimal dosing and treatment duration are still undefined, especially in the context of associated mitochondrial toxicity [40], and there is some evidence that linezolid resistance might already be increasing in TB programmes [6, 7]. Susceptibility testing prior to treatment is crucial, as the risk of treatment failure for patients in which undetected resistance is not diagnosed is likely high [7]. A WHO technical consultation in 2017 confirmed critical concentrations for linezolid susceptibility testing on 7H10, 7H11 and MGIT media [23], yet no rapid molecular assay near point-of-care currently exists to detect any of the mutations associated with phenotypic linezolid resistance in either the 23S rRNA gene (*rrl*) or the 50S ribosomal protein L3 gene (*rplC*).

The most commonly reported mutation conferring linezolid resistance is *rplC* C154R [31, 32], which is also the only mutation that met confidence grading criteria for the designation “Associated with Resistance” in the recent WHO mutations catalog [9]. *in vitro* studies of *rplC* C154R mutants have shown linezolid MICs of 4-8 mg/L on MGIT medium [41, 42], which match MIC data from MDR-TB clinical isolates [41–45]. Additional *rplC* mutations (e.g. T460C) [43], along with a variety of *rrl* mutations, including *in vitro* g2299t and g2814t mutants and a clinical a2810c and g2814t double mutant, have also demonstrated marked linezolid MIC increases [23].

### Diagnostic assay design considerations

Linezolid is one of the clearest drug targets for molecular assay inclusion, given that the majority of global phenotypic resistance has been associated with specific mutation “hot spots” in just two genes. Ultimately, any assay for the rapid determination of linezolid resistance should at a minimum include the *rplC* C154R mutation. Although this mutation has an estimated global sensitivity of only 38.2% [9], an assay including this mutation is anticipated to have high real world diagnostic performance given high prevalence of this mutation in settings where the drug is increasingly used [7]. The *rrl* 2270–2299 and 2746–2814 gene regions might also be considered for assay inclusion to potentially improve sensitivity (Supplement) [31].

Developers must also consider the design of potential linezolid resistance detection assays in the context of current regimens to maximize the usefulness of such an assay. As an add-on drug to the longer regimen for treatment of rifampicin-resistant/MDR-TB patients, a low-cost molecular assay to “rule-in” linezolid resistance would be beneficial to rapidly alert clinicians to the presence of resistance and whether they should include an alternative drug in the long regimen. Given the recent endorsement of BPaL and BPaLM regimens [2], it is likely that an assay for detecting resistance to first-line drugs (e.g. isoniazid, rifampicin) in addition to fluoroquinolones, bedaquiline, and/or linezolid would have substantial clinical value to help determine regimen eligibility. Clinicians are currently blind to resistance to many of these drugs (Figures 1 and 2) at treatment start, awaiting pDST results to be returned many weeks into treatment. However, given that global prevalence of linezolid resistance is still low, a “rule-out” assay confirming the absence of resistance-conferring mutations in relevant genes, rather than a “rule-in” test detecting specific mutations, would also be sufficient and have high negative predictive value for the near future to guide regimen selection in most settings. Ideally, developers should pursue a range of both “rule-in” and “rule-out” assays, depending on the parameters of their specific technology of focus, and with the understanding that either assay type would have limitations that would dictate use case depending on clinical context, including local linezolid resistance rates and prevalence of specific linezolid resistance mutations.

## Nitroimidazoles

The nitroimidazoles, including delamanid and pretomanid, are antibacterial compounds that inhibit the synthesis of mycobacterial cell wall components [46]. Delamanid has been recommended by WHO for treatment of rifampicin-resistant/MDR-TB patients on longer regimens [1], with promising treatment outcome data for these regimens to date [47]. Pretomanid, developed by TB Alliance, features in the all-oral BPaL regimen, which was recently recommended by WHO for treatment of MDR-TB (Figures 1 and 2) [2].

### Drug resistance

Nitroimidazole resistance is a concern given evidence to date. Spontaneous mutant studies have suggested a mutation frequency for nitroimidazoles comparable to rates for isoniazid, ethambutol and pyrazinamide [48]. Certain resistance mutations have also been found to occur naturally in the absence of the drugs, raising concerns about baseline resistance. Notably, in the ZeNix trial, 3/181 participants (1.7%) had baseline pretomanid resistance [22]. In Trial 213 of a delamanid-containing MDR-TB regimen, acquired delamanid resistance was documented in 4/341 participants (1.2%) in the delamanid arm compared to 0/170 participants in the placebo arm [49]. As relatively new compounds for TB treatment, it is a priority to identify and characterize all molecular markers of nitroimidazole resistance, though pDST and sequencing are not yet widely established nitroimidazole testing methods [23], and no rapid molecular assay currently exists for resistance detection.

Delamanid and pretomanid are prodrugs that require activation though the F_420_ coenzyme-dependent bioreduction pathway, and so any loss-of-function mutation in F_420_ pathway enzymes, including *ddn* (*Rv3547*), *fgd1* (*Rv0407*), *fbiA* (*Rv3261*), *fbiB* (*Rv3262*), and *fbiC* (*Rv1173*), is expected to result in cross-resistance [50–53]. However, ∼10-17% of phenotypically pretomanid-resistant isolates have no mutations in these genes, suggesting additional resistance mechanisms remain to be discovered [54–56]. Furthermore, the extent of nitroimidazole cross-resistance is still to be fully defined.

There are also many unknowns regarding the frequency and relevance of nitroimidazole resistance mutations [9]. Generally, mutations appear more commonly in *ddn* (12–29%), *fbiA* (15-19%), and *fbiC* (25–55%) compared to *fbiB* (2–4%) and *fgd* (4–7%), though data is limited [8, 54–58]. Given that nitroimidazole resistance mutations span at least five genes and include many indels, rapid resistance test developers face many challenges. With regards to confidence graded mutations, only *ddn* L49P met criteria to receive an interim designation as “Associated with Resistance” to delamanid, with the majority of data obtained from broth microdilution plates that have yet to be WHO endorsed [9]. Additionally, the global prevalence of this mutation in resistant isolates remains unclear. MIC data have highlighted additional resistance mutations (e.g. natural-occurring *ddn* L107P and *fbiA* D49T), though the global prevalence and clinical relevance of these mutations remains undefined [8, 57, 58]. Given that an assay detecting *ddn* L49P would have a theoretical sensitivity of only ∼6.1% for global delamanid resistance detection, more research is urgently needed to characterize nitroimidazole resistance mechanisms, including lineage effects, to ultimately inform assay development.

### Diagnostic assay design considerations

To date, insufficient evidence exists to suggest gene regions, let alone the specific mutations, to be included in rapid molecular assays for nitroimidazole resistance detection. However, in the context of current DR-TB drug regimens, especially BPaL/M, the value of an initial or follow-on assay for nitroimidazole resistance detection is clear. As for all new and repurposed compounds for which the basis of resistance remains undefined, it is recommended that assay manufacturers consider “rule-out” assays for resistance detection, and generally keep their assay designs “open,” covering large swaths or whole genes if possible, as this would add flexibility in downstream interpretation of the assay based upon evolving knowledge of resistance mechanisms, and minimize or avoid expensive and time-consuming assay redesign. In the interim, sequencing can serve to at least confirm the absence of mutations in known nitroimidazole resistance-associated gene regions for this purpose. It is also imperative to invest in sequencing as a research and surveillance tool to define nitroimidazole resistance mechanisms and document prevalence as use of the drugs increases.

## Pyrazinamide

Pyrazinamide is a nicotinamide analogue with unique activity against persister bacilli and synergistic effect in combination with first-line compounds, bedaquiline, and nitroimidazoles, allowing shortening of different regimens (Figures 1 and 2) [59]. For these reasons, pyrazinamide is a key component of both drug-susceptible (DS-) and DR-TB treatment regimens [1]. For the treatment of DS-TB, HRZE, which is a combination of isoniazid, rifampicin, ethambutol and pyrazinamide, is recommended by WHO. The compound also features in the WHO-recommended treatment regimen for mono isoniazid-resistant TB and as an add-on agent for the MDR-TB short– and long-regimens. The compound is also used in combination with bedaquiline, pretomanid and moxifloxacin in the BPaMZ regimen, though insufficient evidence exists to date regarding the safety and efficacy of this regimen to support its use.

### Drug resistance

Given the role of pyrazinamide in TB regimens [60], it is key to assess resistance prior to patient treatment. The prevalence of pyrazinamide resistance has been well documented globally, with rates of 0–25% among DS-TB, and 40–90% for rifampin-resistant/MDR-TB patients, depending on geographical context [61]. Although the risk of patient treatment failure is fairly low if pyrazinamide resistance is not assessed prior to HRZE therapy for DS-TB patients, the risk increases for DR-TB regimens, with one study in Uzbekistan finding over 90% of those with unsuccessful treatment outcomes having undiagnosed, baseline pyrazinamide resistance prior to treatment with a shorter MDR-TB regimen [62]. In a separate study in Quebec, patients with pyrazinamide mono-resistant TB had significantly worse clinical outcomes than patients with fully susceptible strains [63]. Despite relatively high baseline resistance rates in DR-TB populations, diagnostic options are limited, with only the highly complex GenoScholar PZA-TB II (Nipro, Japan) line probe assay receiving conditional WHO recommendation to date [64]. Furthermore, only pDST in MGIT is recommended for pyrazinamide drug resistance detection [23], though results are inconsistent and this method is generally limited to higher-level health centres [65]. Ultimately, given that the GenoScholar PZA-TB II and pDST for pyrazinamide resistance detection are limited to reference laboratories, many patients are treated empirically without confirmation of resistance.

Mutations in the *pncA* gene, encoding pyrazinamidase, account for the vast majority of pyrazinamide resistance. Although *pncA* represents a seemingly ideal single gene target for molecular assays, the resistance landscape of this gene is complicated. First, resistance mutations are spread throughout the length of the 561 bp *pncA* gene and its promoter without clear “hot spots,” or clusters of resistance-associated mutations. Noted resistance mutations include indels and even whole gene deletions [66], and there also exist many neutral polymorphisms not associated with resistance. In the WHO mutations catalog, no fewer than 105 *pncA* polymorphisms were specifically associated with resistance, with many additional mutations having interim resistance associations (i.e. mutations not definitively associated with resistance due to their low frequency) [9]. These factors continue to present significant roadblocks to the design and interpretation of NAATs for rapid pyrazinamide resistance detection at lower levels of the healthcare system.

### Diagnostic assay design considerations

Despite the challenging landscape of pyrazinamide resistance mechanisms, two approaches hold promise for the design of rapid resistance assays. The first is to incorporate all known neutral polymorphisms into a molecular assay and detect any variants different from wildtype, as seen with the Genoscholar PZA-TB II assay. Despite the practical and technical difficulties in assay performance and results interpretation, this design holds promise. In clinical settings, the assay showed a sensitivity of 81.2% and specificity of 97.8% for cultured isolates [67]. In fact, the WHO mutations catalog defined possible expert rules for assay interpretation to further improve test performance for detection of pyrazinamide resistance [9]. However, collective input will still be needed to correlate *pncA* mutations with clinically relevant resistance to aid in resistance diagnosis using these molecular methods.

Alternative approaches, including the assessment of PZase enzymatic activity via assays such as Bioneer AccuPower, have also been suggested as a method to shorten the time to resistance diagnosis. However, these methods either involve days of incubation followed by colorimetric screening [68], or PCR amplification of *pncA* and synthesis of PZase in a cell-free wheat germ protein expression system, from which PZase activity can be assessed by colorimetric methods, which still requires time, technical expertise and external quality assessment [69]. Although these collective approaches remain removed from the point-of-care, they still play a role in resistance detection as well as correlating mutations with phenotypic resistance to clearly highlight key mutations for inclusion in molecular assays.

Notably, in the context of current regimens (Figures 1 and 2), a rapid pyrazinamide resistance assay could have value as either an initial assay used alongside existing isoniazid and rifampicin resistance tests to determine treatment for mono-isoniazid resistant strains (i.e. informing REZLv regimen eligibility), or as a reflex assay to initial isoniazid and rifampin resistance tests to inform eligibility for inclusion in additional regimens (i.e. the MDR-TB short and long regimens). As for bedaquiline and the nitroimidazoles, sequencing should remain a priority for pyrazinamide resistance detection in clinical isolates, as sequencing approaches are well equipped to handle the complex genetic landscape of *pncA*, being able to identify full nucleotide sequences including neutral polymorphisms and indels as well as to filter out non-resistance associated mutations with downstream bioinformatics for accurate clinical interpretation. At a minimum, NGS can “rule out” resistance in the absence of mutations, as well as to inform the field regarding the prevalence of relevant *pncA* mutations and associations with resistance.

## Future technologies and directions

Ideally, diagnostic development should parallel drug development, ensuring that all approved drug compounds are partnered with well-validated resistance assays. However, this is seldom the case, as resistance mechanisms are not fully elucidated for TB drugs even following regulatory approval. During the early phases of drug development, phenotypic susceptibility testing is typically performed against only a small number of isolates, and even in larger clinical trials limited numbers of TB genotypes are generally included [70]. For these reasons, drug trials should make greater efforts to ensure sufficient genotypic diversity in their studies, and that appropriate methodology is employed for detection of phenotypic and genotypic resistance to novel compounds. Furthermore, reporting of these findings must be prioritized to ultimately support the co-development of rapid diagnostics.

The complex resistance landscape and the scarcity of identified mutations and genetic targets associated with resistance has further slowed the development of rapid molecular tests, and only a few novel assays are currently undergoing development and evaluation for the detection of resistance to new and repurposed compounds (Table 1). Sequencing is an important diagnostic solution that can be used to interrogate a wide array of resistance-associated gene regions, but its use is currently limited to central laboratories. Although the first multinational clinical evaluation of culture-free, targeted NGS for DR-TB diagnosis is underway [71], to date the technology has only been WHO recommended for DR-TB surveillance [72]. Ultimately, for most drugs, clinical decision-making remains largely blind to important drug resistance patterns. As the global burden of DS- and DR-TB impacts mostly low-resourced settings [73], development of near-patient, connected molecular technologies should be prioritized with consideration for the fact that even if new drugs or regimens are scaled-up in these countries, there is an increased risk that programmatic impact will not be realized if diagnostic availability and pathways are not optimized for patient management. In order to preserve new treatment compounds and regimens, the development of diagnostics specifically for the detection of linezolid, bedaquiline, nitroimidazole and pyrazinamide resistance for high-burden settings should be a research and implementation priority. In this context, two types of molecular assays should be prioritized: (i) targeted “rule-in” resistance assays covering at a minimum, the resistance associated mutations, and (ii) broad range “rule-out” resistance assays to rapidly detect wildtype vs. “not wildtype” across genes and/or covering multiple genes simultaneously (Table 2), which should have a high negative predictive value while community prevalence is still low.

**Table 2.** Priority molecular diagnostic assays for detection of resistance to new and repurposed compounds in current drug-susceptible and drug-resistant tuberculosis treatment regimens.

Drug	Associated regimens	Resistance prevalence	Risk of treatment failure if additional resistance is not diagnosed	Possible assay approaches	Likely use case	Additional comments
Bedaquiline	BPaL, BPaLM, BPaMZ*, MDR-TB Short Regimen, MDR-TB Long Regimen	Estimates for DR-TB are low (≤4%) [85], but there is increasing evidence of resistance predating drug discovery, and so baseline resistance must be considered.	Moderate to high	Sequencing, rapid molecular assay interrogating whole gene (likely “rule-out” resistance)	Follow on assay to HR result along with M to inform BPaL, BPaMZ* or add-on eligibility in MDR-TB regimens for patients with prior drug exposure (i.e. higher prevalence of resistance) as well as patients with poor response to empiric therapy.	Highest priority drug for inclusion in initial and follow-on tests of resistance along with M. There are additional, baseline resistance concerns for this drug.
Linezolid	BPaL, BPaLM, MDR-TB Long Regimen	Unknown. Likely low (≤4%) for DR-TB [85, 86].	Moderate to high	Targeted rapid molecular assay approach incorporating known mutation “hot spots,” molecular assay interrogating whole gene (likely “rule-out” resistance)**, sequencing	Follow on assay to HR result along with M to inform BPaL, or add-on eligibility in MDR-TB regimens for patients with prior drug exposure (i.e. higher prevalence of resistance) as well as patients with poor response to empiric therapy.	Foreseeable benefit of a single assay generating calls for both M and L as a reflex test to a positive isoniazid and/or rifampicin resistance result, even in absence of information for B resistance.
Pretomanid	BPaL, BPaLM, BPaMZ*, MDR-TB Short Regimen, MDR-TB Long Regimen	Unknown. Likely low for DS-TB but there is increasing evidence of resistance predating drug discovery, and so baseline resistance must be considered. Likely still low (≤4%) for DR-TB [22].	Moderate to high	Sequencing, molecular assay interrogating whole gene (likely “rule-out” resistance)	Follow on assay to HR result along with M to inform BPaL, or add-on eligibility in MDR-TB regimens for patients with prior drug exposure (i.e. higher prevalence of resistance) as well as patients with poor response to empiric therapy.	There are additional, baseline resistance concerns for this drug.
Pyrazinamide	HRZE, REZLv, MDR-TB Short Regimen, MDR-TB Long Regimen	Low to moderate (0-25%) for DS-TB; Moderate to high (40-90%) for DR-TB [61, 86].	Low (for DS-TB), moderate (for DR-TB)	Sequencing, molecular assay interrogating whole gene (likely “rule-out” resistance)	Provides information outside of HR to determine REZLv eligibility for mono-isoniazid resistant TB. Also informs treatment for patients with prior drug exposure and those with poor response to empiric therapy.	Although pyrazinamide testing is unlikely to influence the standard HRZE regimen, this early knowledge can guide treatment for mono-isoniazid resistant TB (i.e. REZLv) and help inform the short– and long MDR-TB regimens.

*Possible future regimen

**If no mutation is identified by a “rule-in” resistance assay, phenotypic testing should confirm sample susceptibility to a given drug.

As molecular TB assays typically interrogate a single, relatively small gene region for any drug (e.g. Truenat RIF and Xpert MTB/RIF and Ultra) [74, 75], developers must prioritize gene regions and mutations for assay inclusion (Supplement). Of the new and repurposed drug compounds, only linezolid currently has the potential for inclusion in a targeted “rule-in” resistance assay, as most associated resistance mechanisms appear to have been well characterized to date. Alternatively, multiple NAATs covering different, diverse genes for one or multiple drugs might be processed in parallel, covering a breadth of resistance mechanisms and theoretically achieving higher sensitivity for a single extraction (e.g. testing multiple Truenat or line probe assays for different gene regions or targets on the Molbio Truelab Quattro or the Hain GT-Blot 48 platforms, respectively). Another option would be to reimagine the targets included in an existing assay such as Xpert MTB/XDR [36]. The removal of de-prioritized injectable compounds from the assay would free up two reporters for resistance detection, and the additional removal of markers appearing at low global frequency, such as *ahpC*, could free up an additional reporter, making room for the three main gene regions associated with linezolid resistance [9, 33]. Ultimately, even with a limited bandwidth for drug resistance detection, such an assay would still be valuable to inform regimen selection by ruling-in linezolid resistance upon mutation detection.

For drugs such as bedaquiline, the nitroimidazoles, and pyrazinamide, a “rule-out” resistance approach is preferred to profile large, complex genes with a wide variety of mutations. However, it should be noted that current assays capable of amplifying and interrogating an entire gene region (e.g. Genoscholar PZA-TB II), as necessary for drugs such as pyrazinamide and bedaquiline, have limitations such as difficulty in visual reading, interpretation of indels, detection of heteroresistance, and differentiation of non-resistance-conferring mutations. Although sequencing is well-situated to address some of the limitations of existing molecular assays as “rule-in” and/or “rule-out” resistance tests, the need for a prior culture step for whole genome workflows limits accessibility and utility of the technology for lower-level healthcare centres. While tNGS increases the utility of sequencing for drug resistance detection in complex targets and does not require culture or biosafety level 3 facilities, these technologies are still limited to higher-level healthcare centres (Figure 2) due to workflow complexity and sample batching requirements to achieve affordability. For broader application of sequencing methods, workflow simplification must be a top priority [76].

Regardless of what solution is pursued for development, developers should also be mindful of the needs and resources available to higher-level reference laboratories and lower-level healthcare centres. Notably, test results should be suitably rapid with integrated data analysis to enable same-day decision making, with price commensurate with the number of, and importance of, included drugs to aid in regimen selection in line with target product profiles for next-generation drug susceptibility tests and NGS [77, 78]. More practically, low complexity tests should be designed to be operated by users with minimal technical experience and training, also ensuring that sample type and sample preparation for testing are not major hurdles to implementation. Operation and storage parameters should match settings of intended use [77]. In addition, the greater emphasis being placed on TB preventive therapy necessitates broader integration of molecular diagnostics to support decision-making.

In order to further propel diagnostic development for novel and repurposed compounds, the following research areas should be prioritized (Table 3). First, given that resistance mechanisms for these drugs have not been frequently identified in clinical studies [9], there is a need for a strong repository of clinical isolates with associated phenotypic and genotypic data. Routine reference laboratories performing pDST as well as those participating in clinical studies, especially studies of TB patients undergoing treatment follow-up, should consider banking samples and sequencing isolates to grow the collective knowledge of drug resistance mechanisms as per the WHO public call for data [38], and to make these samples available for assay development efforts. This is especially true of evaluations with clinical outcome data. Second, data from country-led operational research and surveillance efforts, especially data collected when rolling out new TB drugs and regimens, should be collated to inform diagnostic development in the context of patient-management. Third, the validation of microtiter DST plates, as well as broader use of whole genome sequencing in DR-TB surveillance, will also help identify phenotypic-genotypic associations for new drugs as well as frequencies of resistance-associated mutations. Finally, research efforts should focus not only upon binary phenotypic and genotypic data, but also on results of allelic exchange and enzymatic activity experiments, and consider epistasis and lineage-effects when undertaking any studies of resistance mechanisms, to better interpret the role of borderline resistance mutations as well as host-associated factors, such as NAT-2 polymorphisms, to guide drug dosing during treatment [79]. Together, these efforts alongside ongoing work to enrich the mutations catalogue are anticipated to yield strong data for newer drugs and strengthen resistance association evidence for mutations, thus aiding technology development. Additionally, it is recommended that diagnostic development efforts specifically tailored for low-resource settings are prioritized [77]. Only with these design, development and implementation considerations can rapid molecular diagnostics for novel drugs be truly useful and fit-for-purpose at all levels of the healthcare system.

**Table 3.** Current challenges to the design of molecular assays for resistance detection to new and repurposed anti-tuberculosis compounds and research priorities.

Drug	Gene targets (No.)	Challenges for molecular assay inclusion	Research priorities
Pyrazinamide	pncA (1)	• Length of gene• Not all mutations associated with resistance• Indels including whole-gene deletion	• WGS of resistant isolates and phenotypic testing and data synthesis to identify additional, high-confidence resistance mutations • Repository of resistant isolates with phenotypic and genotypic data • Lineage effects
Bedaquiline	pepQ, Rv0678, mmpL5, mmpS5, atpE (5)	• Number of genes • Length of genes • Mutations spread throughout genes • Additional, unknown resistance mechanisms • Not all mutations associated with resistance	• WGS of resistant isolates and phenotypic data synthesis to identify and characterize additional mutations associated with resistance • Determination how results for efflux pump mechanisms may differ before and following drug exposure • Repository of resistant isolates with phenotypic and genotypic data • Lineage effects
Linezolid	rplC, rrl (2)	• Additional, unknown resistance mechanisms	• WGS of resistant isolates and phenotypic data synthesis to identify and characterize additional mutations associated with resistance • Repository of resistant isolates with phenotypic and genotypic data
Pretomanid	fgd1, ddn, fbiA, fbiB, fbiC, Rv2983 (6)	• Number of genes • Length of genes • Mutations spread throughout genes	• WGS of resistant isolates and phenotypic data synthesis to identify and characterize additional mutations associated with resistance • Repository of resistant isolates with phenotypic and genotypic data • Lineage effects

## Conclusion

Despite the availability of large amounts of genetic data from surveillance and other studies, our knowledge of relevant resistance mutations is incomplete for novel and repurposed drug compounds. To date, linezolid presents one of the clearest targets for inclusion in rapid molecular diagnostic assays to “rule-in” resistance, though there are additional resistance mechanisms for this drug that remain uncharacterized. For bedaquiline and the nitroimidazoles, the picture of resistance is more complicated, with several large genes harbouring multiple resistance mutations as well as neutral polymorphisms. For pyrazinamide, although resistance is largely linked to mutations in a single gene, the key challenge is not only covering the entire gene and promoter, but also differentiating specific resistance mutations and indels from non-resistance conferring polymorphisms. As additional data becomes available from large-scale sequencing efforts and microtiter plate studies, the key gene targets for molecular assay inclusion for these drugs will become increasingly apparent. In the meantime, solutions such as assays incorporating all known neutral polymorphisms into a molecular assay and detecting any variants different from wildtype are needed now to rapidly direct patient treatment, especially at lower levels of the healthcare system where the majority of patients enter the care cascade. In parallel, sequencing should be considered whenever available for resistance detection, using a similar “rule-out” resistance approach to inform treatment algorithms while also building the knowledge base of relevant resistance mechanisms for new and repurposed drug compounds. Furthermore, the increased capacity built for sequencing as a result of the COVID-19 pandemic should be exploited to promote tNGS approaches for DR-TB diagnosis at an intermediate healthcare level.

## Supplementary Material

Supplemental Material
